# Cartilaginous Choristoma of the Tonsil: Three Case Reports

**Published:** 2015-07

**Authors:** Recep Bedir, Özlem Celebi Erdivanli, Başar Erdivanli, İbrahim Sehitoglu, Engin Dursun

**Affiliations:** 1 *Department of Pathology, Recep Tayyip Erdogan University of Medical Faculty, Rize, Turkey.*; 2 *Department of Otorhinolaryngology, Recep Tayyip Erdogan University of Medical Faculty, Rize, Turkey.*; 3 *Department of Anaesthesiology and Reanimation, Recep Tayyip Erdogan University of Medical Faculty, Rize, Turkey.*

**Keywords:** Fibrocartilage, Choristoma, Palatine tonsil

## Abstract

**Introduction:**

Choristoma is defined as the presence of cells in abnormal locations due to defects during embryological development. The word choristoma implies a neoplasm; whereas heterotopia refers to a displaced tissue without necessarily being a swelling or a neoplasm. Literature contains reports of cartilaginous choristoma in the cervix, endometrium, breast tissue, and oral region.

**Case Reports:**

Three cases of cartilaginous choristoma, which were accidentally found during microscopic examination of excised tonsil tissues, are presented.

**Conclusion:**

Choristomas may cause difficulty in the differential diagnosis of true neoplasms, since they are rare and may grow. Therefore pathologists should be considered in the differential diagnosis of cartilaginous lesions, because cartilaginous choristomas of the tonsil are a rare entity.

## Introduction

Choristomas are benign lesions, rarely seen in head and neck region. They were also reported in the pharynx, hypopharynx, oral mucosa, and middle ear. Osseous and cartilaginous choristomas occur more commonly in the dorsum of the tongue (1). The oral cavity may harbor cartilage, glial tissue, salivary glands, gastric mucosa, bone, thyroid tissue, and sebaceous glands (2,3). A choristoma of the tonsil is very rare; to date, less than 10 cases were reported. Three cases of choristoma of the tonsil, which were accidentally found during microscopic examination of excised tonsil materials, are presented

## Cases Report

The first patient was a 50-year-old male, who displayed recurrent tonsillitis, and hypertrophy of the right tonsil (tonsillar hypertrophy grading scale +4/+1). The excised tonsil was a 4 x 2 x 1.8 cm wide, hard, solid lesion. The second patient was a 4-year-old male, who displayed snoring, sleeping with the mouth open, and recurrent tonsillitis. Physical examination showed adenoid vegetation obstructing 75% of the choana and tonsillar hypertrophy (+3/+3). During adenotonsillectomy, right (2.5 x 2x 1 cm) and left (2.5 x 1.8 x 1 cm) tonsils were excised. They were soft and solid tissues.

The third patient was a 28-year-old male, who displayed recurrent tonsillitis. Tonsils were hypertrophic (+3/+3). Following bilateral tonsillectomy, right (3.2 x 2 x 1.5 cm) and left (3 x 1.8 x 1.5 cm) tonsils were excised. They were soft and solid tissues.

**Fig1:**
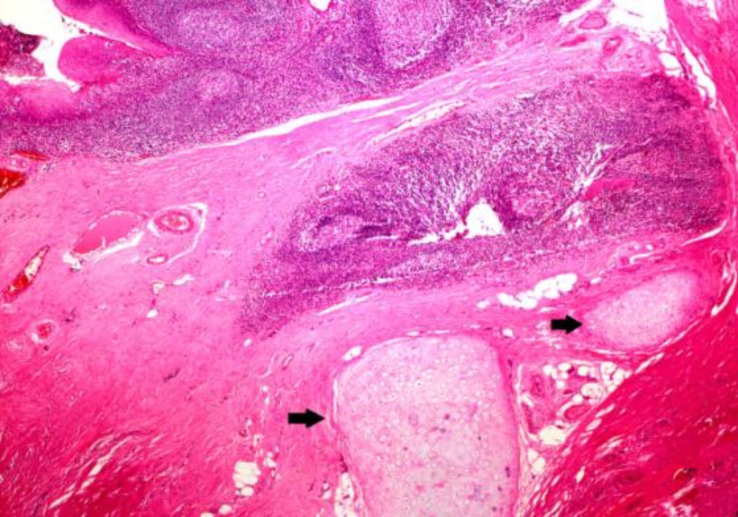
Islands of mature cartilage were seen embedded in the fibrocollagenous tissue adjacent to follicular hyperplasia in the tonsil, Case 1 (H&Ex, 40)

**Fig 2:**
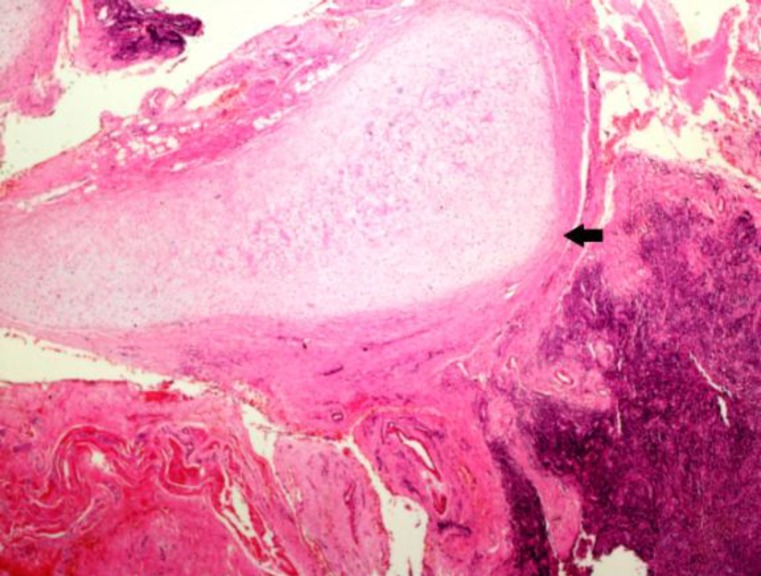
Island of mature cartilage (arrow black) was seen embedded in the fibrocollagenous tissue, Case 2 (H&Ex, 40)

**Fig 3:**
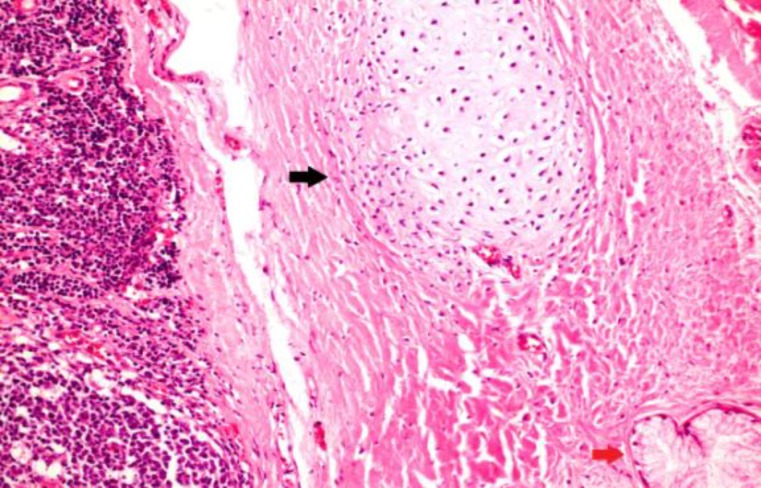
Hyaline cartilage (arrow black) and minor salivary glands (red black) were seen in the tissue around the tonsils, Case 3 (H&Ex, 200

In all cases, the tonsils’ surface was greyish brown and the inner part was light brown. Conventional light microscopic examination of tissues showed signs of chronic tonsillitis characterized by reactive lenfoid hyperplasia with distinct germinal centers under multi-columnar stratified squamous epithelium and mature hyaline cartilage islands within the soft tissue around lenfoid follicules (Figs.1-3).

**Fig 4:**
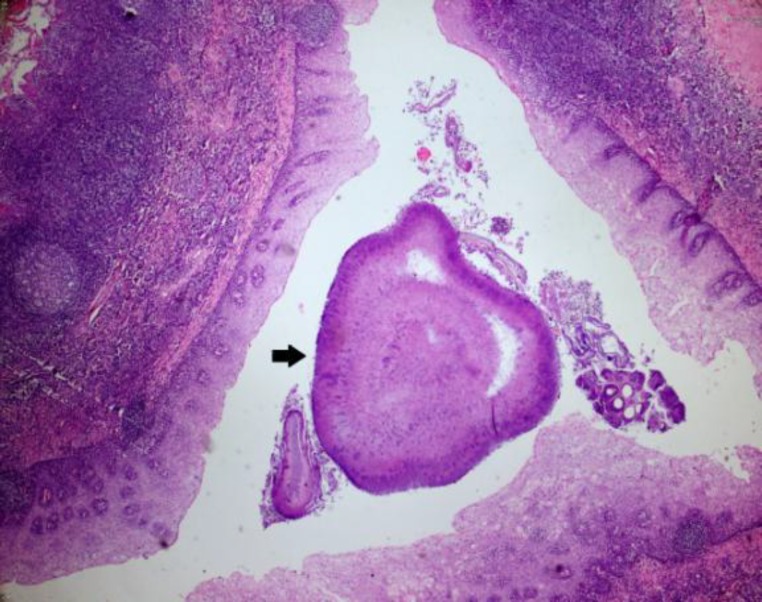
Actinomyces colonies were observed in the mucosa of the tonsil surface (H&Ex, 200)

Also, epidermal inclusion cysts in the superficial mucosa and actinomyces colonies were present in the tonsil tissue (Fig. 4).

## Discussion

Embryological anomalies are common in the neck region due to its complexity. Cartilaginous choristoma may be a developmental anomaly due to its common occurrence in the head and neck region. It was first defined by Berry in 1890. The age of the diagnosis for these patients varied greatly, ranging from 10 to 80 years (3,4). Cartilaginous choristomas are characteristically painless, hard nodules found in young females. They are reported in several locations in the head and neck region; however, they are more frequently seen in the oral cavity (5). They are found during histopathological examination of exised tonsils due to chronic tonsillitis. Erkilic et al. reported a 3% incidence of cartilaginous choristoma on tonsillectomy specimens (6).

Cartilaginous choristomas should be differentiated from cartilaginous metaplasia. In the oral cavity, cartilaginous metaplasia is usually seen in soft tissue under poorly fixed dentures. It is histopathologically character- rized by diffuse dystrophic calcification zones and single or clustered cartilage cells at different stages (7).

There are several hypotheses for the pathogenesis of choristoma. Haemal et al. suggested differentiation of multi-lineage mesenchymal progenitor cells (8).

Lindholm et al. suggested formation of osteogenic materials due to chronic inflammation (9), induced bone formation, and heterotopic cartilage proliferation. Growth of multipotential mezenchymal cells may be stimulated by inflammation, trauma, or irritation. Such de novo lesions may seldomly appear in the nasopharynx (3). 

Partihiban et al. suggested that choristomas of the tonsil are related to a developmental anomaly of the second pharyngeal arch and may cause recurrent tonsillitis (10). Tonsils develop from the lateral part of the second pharyngeal arch. An anomaly during the development may cause abnormal mezenchymal tissue in the tonsil (5).

Proper treatment is simple excision of the lesion with the surrounding tissues. No recurrence of cartilaginous choristoma was reported in the head and neck region. However, in other regions, the perichondrium should be excised too, due to risk of new cartilage formation and hence recurrence. These lesions are benign as in any other cartilaginous tissue (1).

## Conclusion

Choristomas may cause difficulty in the differential diagnosis of true neoplasms, since they are rare and may grow. Therefore the pathologist should be aware of the properties of such lesions to avoid false diagnosis.
